# Social interaction and gender as factors affecting the trajectories of children's engagement and hyperactive behaviour in preschool

**DOI:** 10.1111/bjep.12383

**Published:** 2020-10-17

**Authors:** Madeleine Sjöman, Mats Granlund, Anna Karin Axelsson, Lena Almqvist, Henrik Danielsson

**Affiliations:** ^1^ School of Learning and Communication CHILD Research Group Jönköping University Sweden; ^2^ Malmö University Sweden; ^3^ Mälardalen University Sweden; ^4^ Linköping University Sweden

**Keywords:** preschool, trajectories, hyperactive behaviour, engagement, peer‐to‐child interaction, teacher responsiveness

## Abstract

**Background and aims:**

Social interactions in preschool and a child's gender are, in cross‐sectional studies, related to the child's overall levels of hyperactive behaviour and engagement in preschool activities. However, whether social interaction and gender can predict children's engagement and hyperactivity is not thoroughly investigated. This study aims to investigate the longitudinal influence of gender, child‐to‐child interaction, and teacher responsiveness on the association between trajectories of children's levels of core engagement and hyperactive behaviour. It was hypothesized that peer‐to‐child interaction and teacher responsiveness in preschool settings are related to positive change in engagement among children with hyperactive behaviour, especially for boys.

**Sample and methods:**

Swedish preschool staff completed questionnaires assessing the variables of interest for children aged 1–5 (*N* = 203). Data were collected on three occasions over a two‐year period. Latent growth curve (LGC) models were used to explore whether teacher responsiveness, peer‐to‐child interaction, and gender predict trajectories of engagement and hyperactivity.

**Results:**

The results revealed that high levels of hyperactivity were associated with lower levels of engagement on the first occasion. Positive peer‐to‐child interactions and responsive teachers were significant predictors of an increased level of engagement and decreased level of hyperactive behaviour, especially for boys.

**Conclusions:**

The findings underscore the need to improve social interactions, especially peer‐to‐child interactions, to improve engagement in children with hyperactive behaviour, especially boys. Implications for practices and research are discussed.

## Background

Much attention has been paid to the importance of children's engagement in everyday activities in early childhood as essential for their development, learning, and well‐being (Searle, Miller‐Lewis, Sawyer, & Baghurst, 2013). Level of engagement, or focus of attention, is considered the most important predictor for successful learning outcomes for children in general (Appleton et al., 2008) and for children in need of special support (Carpenter et al., 2015). Preschool is an environment where many children enter their first formal peer group, which places high demands on engagement related to abilities such as cognitive self‐regulation and social skills (Allan, Allan, Lerner, Farrington, & Lonigan, 2015). Hyperactive behaviour more commonly found among boys than girls during preschool years (Searle et al., 2014; Walker, Berthelsen, & Irving, 2001) is reported to be negatively associated with engagement (Sjöman, Granlund, & Almqvist, 2016). Low engagement combined with hyperactive behaviours probably makes children more vulnerable in terms of learning and well‐being, both short and long term (Sabol et al., 2018; Fantuzzo et al. 2003; Fantuzzo et al., 2007). Earlier cross‐sectional studies, however, show that social interactions play a crucial role in supporting the engagement of children with hyperactive behaviour, especially for boys (Bulotsky‐Shearer, Bell, & Dominguez, 2012; Hill, Degnan, Calkins, & Keane, 2006).

Although research shows an association between children's low engagement and hyperactive behaviour in preschool, the mechanisms that may help explain these associations are unclear. This study investigates how social interactions and gender affect the trajectories of engagement and hyperactive behaviour in preschool children.

### Engagement and hyperactive behaviour in early childhood

#### Definition of engagement

Engagement has been defined as the amount of time the child is actively involved with materials or other persons or in a situation including activities at different complexity (De Kruif & McWilliam, 1999). Researchers have focussed either on the intensity of the child's involvement in learning activities (Drake, Belsky, & Fearon, 2013; Skinner, Furrer, Marchand, & Kindermann, 2008) or on engagement in activities at different complexity (Aguiar & McWilliam, 2013; Raspa, McWilliam, & Maher Ridley, 2001). Engagement in complex activities, such as make‐believe play (De Kruif & McWilliam, 1999), is more common among older children and seems to be related to their cognitive development (Piaget, 1962; Vygotskij & Cole, 1978).

In more recent research, however, it has been possible to distinguish between two kinds of engagement; developmental engagement and core engagement (Sjöman et al., 2016). Developmental engagement is related to the complexity of the activity the child engages in and thus is related to levels of cognitive development (Aguiar & McWilliam, 2013), core engagement refers to the overall attentional and persistent behaviour in activities, independent of the complexity of the activity (Sjöman et al., 2016). It has been found to be associated with social interaction in preschool (Ibid) and academic achievement in school (McClelland, Acock, Piccinin, Rhea, & Stallings, 2013). Because of the weak relationship with the complexity of the activity the child participates in, core engagement has only a weak statistical relationship to children's chronological age (as a proxy for maturity) (Pierce‐Jordan & Lifter, 2005; Sjöman et al., 2016).

Independent of child age and maturity, studies have demonstrated that core engagement (i.e. attentional and persistent behaviour) is associated with children's learning (Cadima, Doumen, Verschueren, & Buyse, 2015; Ladd, & Dinella, 2009; Searle et al., 2013; McClelland et al., 2013). Specifically, a longitudinal study by Mokrova et al. (2013) shows that children's motivation at the age of three, operationalized as persistent behaviour in completing a challenging task, predict their academic achievements in Kindergarten. Similarly, a longitudinal study by Claessens and Dowsett (2014) indicates that improvements in attentional behaviour during preschool predict children's achievement gains through third grade. Children exhibiting hyperactive behaviour, however, are at greater risk for low core engagement and learning difficulties that may have an impact on achievement in elementary school. More research is needed about how hyperactive behaviour and core engagement influence each other over time. Knowledge of how to increase children's core engagement in preschool has implications for society's possibilities to support children's later school success, especially for children with hyperactive behaviour.

#### Definition of hyperactive behaviour

Hyperactive behaviour definitions contain two dimensions that can be observed as being constantly overactive, cannot stay still for long, and as short attention span, respectively. In most measures of hyperactive behaviour, for example Strength and Difficulty Questionnaire (SDQ: Goodman, 1997), used the present study, hyperactive behaviour is represented both by items assessing being overactive and inattentive respectively (Doernberg and Hollander, 2016). This definition is based on the DSM 5 definition of ADHD (American Psychiatric Association, 2013). However, in this study the focus is on hyperactive behaviour as a continuum from low to high, not only children with a cut‐off score for exhibiting high hyperactive behaviour. Preschool requires that the children can initiate, maintain, and finish activities in a socially accepted manner, which requires sustained attention and persistent behaviour (Spira & Fischel, 2005). A systematic review by Allan et al. (2015) showed that hyperactive behaviour is linked to lower levels of inhibitory control and lack of flexibility. This can have a negative influence on children's ability to adapt to a challenging environment such as preschool.

The trajectories of hyperactive behaviour are, over time, related to several other behavioural difficulties, such as conduct problems and poor social functioning (Gustafsson et al., 2018). However, over time hyperactivity tends to decrease for most children although some children continue to exhibit high levels (Ibid). In severe cases, hyperactive behaviour in early ages might be an early marker for a neuropsychiatric diagnosis later in life (Hong, Tillman, & Luby, 2015; Smith et al., 2017), such as attention deficit hyperactivity disorder (ADHD; American Psychiatric Association, 2013).

If hyperactive behaviour is seen as a continuum from low to high it is essential to investigate hyperactivity in all children, not only those above clinical cut‐off points for having hyperactivity problems (Bremberg & Dalman, 2015). Further, the covariation between hyperactive behaviour, engagement, and gender needs to be studied over time.

### Trajectories of engagement and hyperactive behaviour: Potential gender differences

Since several studies indicate higher engagement in preschool activities for girls compared with boys (Searle et al. 2014; Hoang et al., 2019; Walker, Berthelsen, & Irving, 2001) and hyperactivity is more prevalent among boys, gender is an essential characteristic to consider when studying the trajectories of children's level of core engagement and hyperactive behaviour. For instance, a comparison study conducted in Israel, Netherlands, and Finland showed a higher level of on‐task behaviour in preschool settings for girls compared with boys in all countries (Brody et al. 2020). In addition, gender differences in behavioural problems were also found in a recent population‐based intervention trial conducted in Swedish preschool settings (Dahlberg et al., 2019). More specifically, both teachers and parents rated higher scores in hyperactive behaviour for boys compared with girls, using the Strengths and Difficulties Questionnaire (SDQ; Goodman, 1997). On the other hand, a prospective longitudinal study (Smith et al., 2017) showed no gender‐related differences in hyperactive behaviour at age three but a significant gender‐related difference in long‐term effects. Hyperactive behaviour in boys during preschool years predicted poor mental outcomes in youth, which was not the case for girls.

How gender differences in hyperactive behaviour affect engagement over time is however unclear. Two studies (Sawyer et al., 2015; Searle et al., 2014) conducted in preschool settings, where proxy ratings were used revealed that boys exhibiting high levels of hyperactivity/inattention showed significantly lower engagement, compared to girls with high levels of hyperactivity/inattention. The longitudinal impact of gender in engagement and hyperactive behaviour needs to be further investigated.

### Social interaction as a predictor for engagement and hyperactive behaviour

Both cross‐sectional and longitudinal studies (Fuhs, Farran, & Nesbitt, 2013; Sjöman et al. 2016; Spivak & Farran, 2016) show an association between classroom processes and less behavioural difficulties and a high level of involvement. This implies that contextual factors, such as peer‐to‐child interaction and teacher responsiveness, that might influence the trajectories in hyperactive behaviour and engagement need to be further investigated.

Positive social interactions, as indicated by teacher responsiveness and positive child–child interactions (Bronfenbrenner & Evans, 2000; Hamre, Hatfield, Pianta, & Jamil, 2014; Sabol & Pianta, 2012), have shown to be important for engagement. Teacher responsiveness is characterized by the use of positive feedback and positive emotional tone in interacting with the child, aspects that have been found to promote children's engagement and development (Cadima, Verschueren, Leal, & Guedes, 2016; Ladd, & Dinella, 2009). Externalizing behavioural difficulties are perceived by teachers as a source of stress (Greene, Beszterczey, Katzenstein, Park, & Goring, 2002) that might lead to teachers' adverse reactions towards the child (Birch & Ladd, 1998). Such reactions might increase the frequency of conflict in the relationships between teachers and children (Mejia & Hoglund, 2016) and have been linked to decreases in teachers' self‐rated responsiveness (Almqvist, 2006a). Decreased responsiveness might negatively affect children's engagement. Improvement in positive peer relations such as peer acceptance, number of friendships, and proximity to others is a mediating factor for decreasing hyperactive behaviour. This effect seems to be particularly strong for boys (Witvliet, Van Lier, Cuijpers, & Koot, 2009). Accordingly, both gender and social interactions are associated with hyperactive behaviour and engagement. However, more research is needed of how gender and social interaction predicts children's engagement and hyperactive behaviour over time. It may be that functional social interactions with teachers and peers are predictors of hyperactive behaviour on engagement, and such interactions might differ between boys and girls.

### Purpose and hypotheses

This study aimed to investigate the longitudinal association of gender, peer‐to‐child interaction, and teacher responsiveness on the association between trajectories of children's core engagement and hyperactive behaviour. Data from Swedish mainstream preschool units involving children, aged 1 through 5, with and without need of special support, were used. The following four hypotheses were tested:

1. High levels of core engagement in children will be associated with low levels of hyperactive behaviour, both at T1 and over time.
2. High levels of teacher responsiveness and peer‐to‐child interaction will be associated with high levels of core engagement and low levels of hyperactive behaviour, both at T1 and over time.
3. Girls will display higher levels of core engagement at T1 and a less steep increase of core engagement over time than boys.
4. Girls will display lower levels of hyperactive behaviour at T1 and a steeper decrease in hyperactive behaviour over time than boys.

## Methods

The present longitudinal study was conducted in preschools for children aged 1‐5. The data are based on surveys answering by preschool staff and were collected during the fall, between September and December, at three occasions over a two‐year period, 2012–2014.

### Participants

The sample for the present study is 203 children—114 boys and 89 girls—between the ages of 18 and 71 months. The preschools were placed in the mid‐south to south‐east region of Sweden, with units in large municipalities (>200.000 inhabitants), smaller or rural municipalities, or units in, or close to, mid‐size municipalities. Data about the children were collected during the fall on three different occasions over a two‐year period. As Table 1 shows, the sample included children with and without need of special support and children who were early second language learners of Swedish (EL2). 'Children in need of special support' is here defined as children with a formal diagnosis (e.g. autism) and/or children informally assessed by staff as having behavioural difficulties (e.g. hyperactive behaviour) affecting their everyday functioning in preschool (Table 1).

**Table 1:** Children's demographic information and preschool characteristics

	Time point one (T1)	Time point two (T2)	Time point three (T3)
Children's demographics			
Age (months) Mean (range; *SD*)	32 (15–17; 9.05)	44 (24–69; 9.13)	55 (36–71; 8.88)
In need of special support, *n* (%)	40 (20)	42 (21)	45 (22)
EL2, *n* (%)	48 (24)	50 (25)	52 (26)
Preschool characteristics			
Group size *M* (range; *SD*)	20 (9–44; 8.81)	21 (10–45; 7.6)	25 (13–47; 10.0)
Number of children in need of special support Range (*SD*)	0–9 (.96)	0–6 (1.4)	0–4 (1.15)
Number of children with EL2 Range (*SD*)	0–40 (12.0)	0–40 (10.6)	0–41 (10.5)

Note

EL2 = Early second language learners of Swedish.

The preschool staff rated children's behavioural difficulties with the Strength and Difficulties Questionnaire (SDQ) developed by Goodman (1997). According to the recommended cut‐off scores in SDQ (ibid), 141 children (81 boys and 60 girls) demonstrated one or more behavioural difficulties, described above, at one or more points in time (see Table 2) or exhibited behavioural problems that negatively affected the child's everyday functioning in the preschool setting.

**Table 2:** Numbers of children displaying different types of behavioural difficulties according to SDQ and percentages of the total sample these represent

	T1	T2	T3
Emotional symptoms scale *n* (%)	5 (2.5)	5 (2.5)	4 (2)
Conduct Problems Scale *n* (%)	55 (27)	45 (22)	68 (33)
Peer problems scale *n* (%)	29 (14.5)	17 (7.8)	7 (3.5)
Hyperactivity scale *n* (%)	28 (14)	35 (17.2)	28 (13.8)

Note

The proportion of behavioural difficulties is displayed separately for each scale. Children may have more than one type of behavioural difficulty.

The child–teacher ratio in classrooms for toddlers (i.e. children between 15 and 36 months) was on average 5:1 (*SD* = 1.23). The ratio in classrooms for children of preschool age (i.e. those between 37 and 71 months) was 6:1 (*SD* = 1.76).

### Procedures and ethical considerations

Initially, an expert panel consisting of experienced preschool teachers and special educators was consulted to evaluate the questionnaires, which were in Swedish. According to the expert panel's recommendations, some items in the survey were revised to adapt to conditions within the Swedish preschool environment. A more detailed explanation of the revisions is given in the measurements section. A three‐time point longitudinal design was used, with questionnaires filled out by the preschool staff at one‐year intervals.

The ethics committee approved the project in Sweden. Both principals of the preschools and the preschool staff gave written informed consent to participate in the project. The preschool staff informed all parents about the project, and a request for consent to participate was distributed to all parents of children entering preschool at each time point. Each fall between 2012 and 2014, questionnaires were handed out during researchers' first visit to the different preschool units; these were returned during a second visit.

### Measures

The data are based on proxy ratings; that is, preschool staff rated children's social interactions and behaviour, for example, engagement and behavioural difficulties, at each of the three time points.

#### Outcome variables: Engagement and behavioural problems

Children's engagement in preschool was measured with the Child Engagement Questionnaire (CEQ; McWilliam, 1991). The preschool staff rated children's engagement behaviour by free‐recall impressions of the level of each child's engagement with teachers, peers, activities, or materials. The questionnaire consists of 32 items on a four‐point Likert scale. The response alternatives were that the child's behaviour is 'not at all typical' (1), 'somewhat typical' (2), 'typical' (3), or 'very typical' (4). Examples were provided for each item to further clarify the content. For example, for the item 'Seems constantly aware of what is going on around him or her,' the example provided is *The child looks at the source of noises and at moving objects and people*. In the present study, only 29 of the original 32 items were used since feedback from the expert panel indicated that three of the items were not appropriate for the Swedish preschool context. For instance, the example provided for the item 'Uses repetitive vocalizations' is *The child says, 'Ba‐ba‐ba‐ba‐ba*'. This type of engagement behaviour is more frequently observed in infants, who, in Sweden, are usually cared for at home during their first year of life. Earlier studies have reported good content and construct validity, as well as intra‐rate reliability, for CEQ (Almqvist, 2006b; Sjöman et al., 2016).

Following the study by Sjöman et al. (2016), the measures in CEQ consist of two underlying constructs. The first, core engagement, is primarily a rating of focus of attention/less complex behaviour (e.g. the child pays attention to what is going on around him or her or attempts to solve a problem even if it takes a long time to finish); this construct has a relatively low correlation with chronological age (*r *= .28). The second construct, developmental engagement, is primarily related to the complex activities (e.g. the child pretends toys are something else when he or she opens a present, and he or she tries to play with an unfamiliar toy without adult help); this construct has a higher correlation with chronological age (*r* = .54). The two constructs explained a total of 52% of the variance, explained in more detail in Sjöman et al. (2016).

Since the purpose was to explore whether social interactions predict trajectories of children's engagement regardless of chronological age or developmental level, only the 11 items representing core engagement (e.g. tries to get adults to do things, watches or listens to other children, seems constantly aware of what’s going on around him or her) were used in the analyses. In the current study, the Cronbach alpha coefficient for Core engagement for each time point was .88, .88, and .86.

#### Behavioural difficulties

Children's behavioural difficulties were measured using the SDQ (Goodman, 1997). The instrument consists of 25 items covering five subscales related to conduct problems, hyperactive behaviour, emotional problems, peer problems, and prosocial behaviour, rated on a three‐point Likert scale, from 'not at all' (0) to 'only a little' (1) or 'quite a lot' (2). The total score on the behavioural difficulties scale is divided into three subgroups—normal, abnormal, and borderline—using cut‐off scores for each subscale (Goodman, 1997). In the analysis of the present study, only the scale measuring hyperactive behaviour with five items was used, with the total scores ranging between 0 ('no hyperactive behaviour') to 10 ('high level of hyperactive behaviour'). The SDQ preschool version has been validated for Swedish preschools (Gustafsson, Gustafsson, & Proczkowska‐Bjorklund, 2016). The internal consistency for the subscale of hyperactive behaviour at each time point was α = .85, .89, and .89.

#### Predictors: social interactions in preschool

Social interactions were measured by using the questionnaire Your Child, Your Interaction (Granlund, & Olsson, 1998), in which preschool teachers rated their experiences of different types of social interactions with a child in the preschool context. The instrument has 36 items covering interactions between teacher and child, child and teacher, peers and child, and child and peers. The responses are based on a five‐point Likert scale, where 1 = 'seldom' and 5 = 'often'. In the analysis of the present study, 15 items were used for two subscales measuring teacher responsiveness to the child (10 items) and other children's interaction with the child (five items). The internal consistency for the two subscales at each occasion was α = .75, .80, and .71 for teacher responsiveness, and α = .92, .90, and .91 for other children’s interaction with the child.

### Missing data

On average, there were 1.1% missing data over all items. SDQ had most missing data, but the missingness for the items was different for each time point. The highest missingness was 8 % and was found for item 21 at time point 1, and for item 10 time point 2. In a few cases (max 2% for SDQ at time point 1 and 2), data were missing for all items. A Little MCAR test (Little & Rubin, 2002) indicated missing data at random (MAR). As suggested by Kline (2005), missing data less than 10% can be handled as MCAR or MAR. Therefore, missing data were imputed in r with the package mice (van Buuren & Groothuis‐Oudshoorn, 2011) with the default method (predictive mean matching). Data for individual items were imputed based on the other items at the same time points and when all items were missing, data were imputed based on all other variables. One complete (imputed) data set was saved and used in further analyses.

### Data analysis

The data were analysed in AMOS 21.0 (Arbuckle, 2012). Due to non‐normal distribution for core engagement at time point two (T2) (Skewness, −1.23; Kurtosis, 1.24), and time point three (T3) (Skewness, −1.60; Kurtosis, 2.85) Asymptotically Distribution‐free Estimates (ADS) was most appropriate to use. To explore whether social interactions in the preschool setting are associated with trajectories of core engagement and hyperactive behaviour, latent growth curve (LGC) modelling was used, and two predictors (teacher responsiveness and peer‐to‐child interaction) were added to the models. Gender was added to test whether there were differences between boys and girls in hyperactive behaviour and core engagement at time point one (T1) and in change over time.

The first step in the analysis was to investigate the within‐person trajectories, that is the direction and extent to which girls or boy’s estimation in hyperactive behaviour and core engagement changes from T1 to T3. The trajectories of core engagement and hyperactive behaviour were examined separately with unconditional growth models with three time points (i.e. baseline, 12 months, and 24 months). Initial models had intercepts with equal path weights, linear slopes with paths 0, 0.5, and 1, respectively, and the same error for the three measures. These assumptions did not hold for either core engagement or hyperactive behaviour. Therefore, different models were explored for core engagement and hyperactive behaviour, respectively. It was found that the models where the growth rate was not equal between time points (i.e. the path between slope and time point two was set free) and the errors for core engagement could differ. That is, error for the measures T3 of core engagement and of hyperactive behaviour, respectively, differ from the other errors. In addition, two covariances between error terms for core engagement and hyperactive behaviour T1 and T2 were added. The modification resulted in a model with a good fit, and thus, this model was used for hyperactive behaviour and core engagement in the subsequent analyses. In the second step, models for core engagement and hyperactive behaviour with a good fit were combined into a single unconditional multivariate model, in which covariance between the intercepts and the slopes for core engagement and hyperactive behaviour respectively were allowed. In the third step, two time‐invariant predictors related to environmental factors, that is teacher responsiveness and peer‐to‐child interactions, were introduced with paths to both intercept and slope for core engagement and hyperactive behaviour. Finally, gender was added as a time‐invariant predictor with paths to both intercept and slope for core engagement and hyperactive behaviour respectively, to investigates differences for boys vs. girls.

Three indices for model fit were used: *X* ^2^, comparative fit index (CFI; Bentler, 1990), and root mean square error of approximation (RMSEA; Browne & Cudeck, 1993). For *x^2^*, *p *> .05 was used as the criterion for a good model fit; that is, no differences between the model and the data. Comparative fit index values above .90 indicate a good model fit (Byrne, 2013). Root mean square error of approximation values less than .05 indicate a very good model fit and values between .05 and .08 indicate a moderate model fit (Browne & Cudeck, 1993). All these criteria had to be met for a model to be considered a good fit.

## Results

Initially, the results present descriptive data for variables of interest. After that, the results from the hypothesis testing are described in the final conditional model, where all the variables are included (hyperactive behaviour, core engagement, peer‐to‐child interaction, teacher responsiveness, and gender).

### Descriptive results

Peer‐to‐child interaction and teacher responsiveness were examined to determine linear stability in core engagement and hyperactive behaviour. In addition, correlations among variables of interest and demographic variables were analysed (Table 3).

**Table 3:** Means, standard deviations, and correlations for core engagement and hyperactive behaviour at T1–T3, and time‐invariant predictors: peer‐to‐child interaction (T1), teacher responsiveness (T1), and child's gender

	1	2	3	4	5	6	7	8	*M*	*SD*	Skewness	Kurtosis
1. Gender	–											
Core engagement												
2. T1	−.059	–							3.40	.55	−.765	−.275
3. T2	.13	**.51** **	–						3.53	.52	−1.23	1.24
4. T3	.23**	**.20** **	**.43** **	–					3.64	.45	−1.60	2.85
Hyperactive behaviour												
5. T1	−.029	−.47**	−.29**	−.21**	–				.61	.49	.96	.38
6. T2	−.17*	−.42**	−.53**	−.28**	**.47** **	–			.57	.56	1.05	.20
7. T3	−.24**	−.25**	−.44**	−.55**	.**50** **	**.56** **	–		.46	.51	1.11	.46
Social interactions												
8. PI	−.03	.58**	.30**	.07	−.41**	−.28**	−.19**	–	3.75	1.01	−.78	−.02
9. TR	.20	.49**	.27**	.09	−.36**	−.32**	−.19**	.51**	4.54	.33	−1.62	4.19

Note

PI = Peer‐to‐child interaction; TR = Teacher responsiveness; hyperactive behaviour, sum score 1–10; core engagement, range 1–4, mean score of 12 items; peer interaction, range 1– 5, mean score of 5 items; teacher responsiveness, range 1–5, mean score of 10 items.

* *p *< .05.

** *p *< .01.

*** *p *< .001

The correlations indicated a significant longitudinal linear stability over time in core engagement (*r *= .20 to .43) and hyperactive behaviour (*r *= .50 to .56). For core engagement, a significant positive correlation between social interactions (peer‐to‐child interaction and teacher responses) and core engagement was revealed for T1–T2. Hyperactive behaviour for T1–T3 was negatively correlated with both types of social interactions. The strongest correlations between T1 and T3 were found between peer‐to‐child interaction, core engagement, and hyperactive behaviour, respectively.

Although high correlations were found between the predictors, teacher–child, and child–child interaction, the collinearity statistics for social interactions for T1 (VIF = 1.351, Tolerance* *= .740), T2 (VIF = 1.356, Tolerance* *= .738), and T3 (VIF = 1.474, Tolerance* *= .679) were within acceptable limits, for example VIF above 10 and Tolerance less .10. Residuals and scatter plots indicated the assumption of normality, linearity and homoscedasticity (Brace, Kemp, & Snelgar, 2009).

### Unconditional model for hyperactive behaviour and core engagement

**Figure 1:**
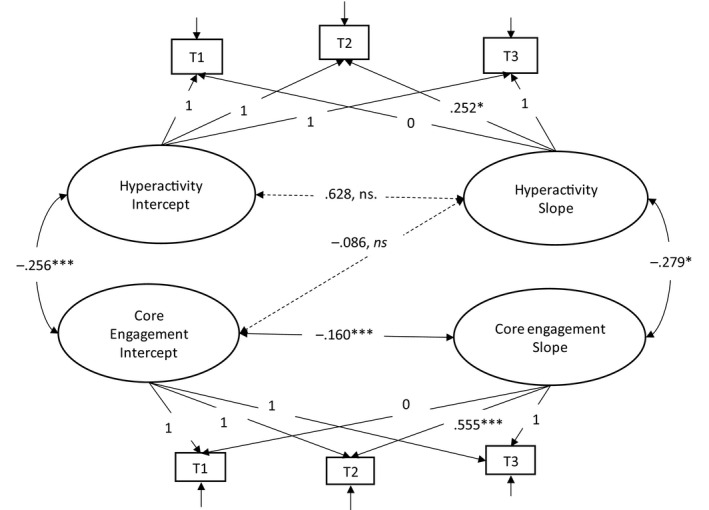
Unstandardized regression weights (B) for unconditional combined model for hyperactivity and core engagement. *Hyperactivity*, sum score 1–10; *Core engagement*, range 1–4, mean score of 12 items. *p < .05; **p < .01; ***p < .001.

The unconditional model for core engagement and hyperactive behaviour (see Figure 1) revealed a poor fit to the sample data (*x* ^2^ (9)* *= 48.508, *p *= .000; CFI* *= .902; RMSEA* *= .147). Thus, the model was optimized (see Data analysis section for further details) by letting free estimation occur between the slope and the error term for time point two of core engagement and hyperactive behaviour, respectively. Two covariates were added between the error terms at T1 and T2 for hyperactive behaviour and core engagement, respectively. The trimmed unconditional model showed an adequate model fit to the sample data (see Table 4).

**Table 4:** The fit indices are provided for the three unconditional models, the conditional model, and the full conditional model. The models contain hyperactive behaviour and core engagement as outcome variables and three predictors; teacher responsiveness, peer‐to‐child interaction, and gender of the child

Models	*x* ^2^	*p*	*df*	CFI	RMSEA (90% CI)
Hyperactive model	.545	.460	1	1.00	.000 (.000–.167)
Core engagement model	1.359	.507	2	1.00	.000 (.000–.124)
Combined unconditional model for hyperactive behaviour and core engagement	5.006	.287	4	.990	.035 (.000–.117)
Full conditional model	8.308	.761	12	1.00	.000 (.000–.051)

* *p *< .05.

** *p *< .01.

*** *p *< .001.

As Figure 1 shows, a non‐significant association was found between the intercept and slope for hyperactive behaviour (*B *= .628, *ns*), indicating that children's hyperactive behaviour at T1 was not associated with change over time. Whereas, a significant correlation was found between the intercept and slope for core engagement (*B *= −.160***), indicating that children's core engagement was associated with change over time. In addition, significant negative association was found between intercept for hyperactive behaviour and core engagement (*B *= −.256***). Also, a significant positive association was found between the slope for hyperactive behaviour and core engagement (*B *= −.279*), respectively. The results indicate that children with low core engagement at T1 had an increase in core engagement over time. For hyperactive behaviour, however, an opposite pattern was found, that is children with higher level of hyperactive behaviour at T1 was not associated with their change over time. In addition, children with larger increase in core engagement also had larger decrease in hyperactive behaviour. The results provided further support for investigating social interaction and gender as predictors for trajectories of hyperactive behaviour and core engagement.

### Latent growth curve analysis

The hypotheses I–IV were used to investigate social interaction and gender as predictors for children's core engagement and hyperactive behaviour at T1 and over time. The conditional model revealed a very good model fit to the data (see Table 4).

**Figure 2:**
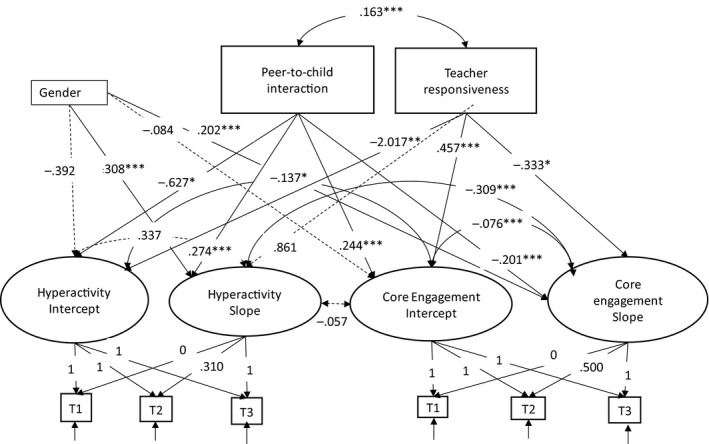
Unstandardized regression weights for final conditional model for hyperactivity and *core engagement* with predictors, *peer‐to‐child interaction*, *teacher responsiveness*, and *gender*. *Hyperactive behaviour*, sum score 1–10; *core engagement*, range 1–4, mean score of 12 items; *peer‐to‐child interaction*, range 1–5, mean score of 5 items; *teacher responsiveness*, range 1–5, mean score of 10 items; *gender*, 0 = boys and 1 = girls. *p < .05; **p < .01; ***p < .001.

The first hypothesis was partly supported, that is, a significant correlation was found between core engagement and hyperactive behaviour, a relatively weak covariance for intercept (*B *= −.137*), meanwhile, a stronger covariance for slope (*B *= .309***) (see Figure 2). In other words, if children displaying attentive and persistence behaviour (e.g. core engagement) at T1 show less hyperactive behaviours. The same pattern was found over time, increase in core engagement was associated with decrease in hyperactive behaviour in children. These paths indicate a covariation for core engagement and hyperactive behaviour at T1 and between the rate of change in core engagement and hyperactive behaviour. However, the non‐significant paths (*B *= −.057, *ns*.) between intercept for core engagement and slope for hyperactive behaviour indicate that children's level of core engagement at T1 did not affect the rate of change in their hyperactive behaviour, nor the other way around: hyperactive behaviour at T1 did not affect the rate of change in core engagement.

The second hypothesis was partly supported (see Figure 2). That is, significant positive paths were found from both types of social interactions (e.g. peer‐to‐child interaction, teacher responsiveness) to the intercept of core engagement (peer‐to‐child interaction: *B *= .244***, and teacher responsiveness: *B *= .457***). At the same time point, negative significant paths were found from both types of social interactions and the intercept of hyperactive behaviour (peer‐to‐child interaction: *B *= −.627***, and teacher responsiveness: *B *= −2.017**). In other words, the results revealed that peer‐to‐child interaction and teacher responsiveness were significant predictors for core engagement and hyperactive behaviour at Time 1. The significant negative estimates between peer‐to‐child interaction and slope for core engagement (*B *= −.201***) indicate that peer‐to‐child interaction was associated to a steeper increase in core engagement for children rated as low in core engagement at T1. Moreover, the significant positive estimates between peer‐to‐child interaction and slope for hyperactive behaviour (*B *= .274***.) indicates a decline in hyperactive behaviour for children rated as high levels of hyperactive behaviour at T1.

Same paths were found for the association between teacher responsiveness and slope for core engagement (*B *= −.333*), which was not the case for teacher responsiveness and slope for hyperactive behaviour (*B *= .861, *ns*). In other words, teacher responsiveness was associated with steeper increase in core engagement for children with low core engagement at T1. This was not the case for hyperactive behaviour for children with high levels of hyperactive behaviour at T1.

The third hypothesis was partly supported. That is, the non‐significant path between gender and intercept for core engagement (*B *= −.085, *ns*.) indicates that there were no significant differences between boys and girls in core engagement at T1. However, as figure 2 shows, a significant difference was found between boys and girls in the rate of change in core engagement (*B *= .292***). In other words, girls (coded 1) showed a less steep increase in core engagement than boys (coded 0).

The fourth hypothesis was partly confirmed. As figure 2 shows, a non‐significant association was found between gender and intercept for hyperactive behaviour (*B *= −.392, *ns*). Whereas, a significant association was found between gender and slope for hyperactive behaviour (*B *= −.917*). Differences were found between girls' (coded 1) and boys' (coded 0) hyperactive behaviour over time, which was not the case at T1. That is, compared with boys, girls showed a steeper decrease in hyperactive behaviour over time.

## Discussion

Framed by the bioecological model (Bronfenbrenner & Ceci, 1994), this study sought to understand social interaction and gender as predictors for trajectories of children's core engagement and hyperactive behaviour. The three main findings were as follows: (1) core engagement is negatively associated with hyperactive behaviour, both at T1 and long term, (2) positive peer‐to‐child interaction is a significant predictor of high core engagement and low hyperactive behaviour at T1. Positive peer‐to‐child interaction is also a significant predictor of a steeper increase in core engagement and predicts a decrease in hyperactive behaviour, (3) high teacher responsiveness is a significant predictor of children's initial level of core engagement and hyperactive behaviour and of a steeper increase in core engagement. Teacher responsiveness do not, however, predict a decrease in hyperactive behaviour.

The results suggest that the contextual factors, level of peer‐to‐child interaction, and teacher responsiveness, contribute to and partly explain trajectories of both core engagement and hyperactive behaviour.

### Stability in core engagement and hyperactive behaviour across preschool years

Our results indicate a significant correlation in core engagement between T1 and T3. Studies have pointed out the importance of children's engagement in preschool for later academic achievement (Cadima et al., 2015; Ladd and Dinella, 2009; Williford, Whittaker, et al., 2013) but without separating core engagement from developmental engagement. Core aspects of engagement do not vary with the complexity of the activity and children's developmental level. Our results indicate a negative association between the rate of change in hyperactive behaviour and core engagement. In other words, increase in core engagement was associated with decreased hyperactive behaviour. Thus, children with hyperactive behaviour might require special support to improve their attention and persistence in everyday activities.

The present results are based on proxy ratings within a continuum of hyperactive behaviour and engagement, from low to high levels, rather than a categorical approach. The results suggest that identification of children in need of special support to a greater extent should be based on a functional approach where everyday functioning is in focus rather than predetermined categories. According to a functional approach, children's difficulties and need for special support are best defined within the context in which they act, for example, preschool (Castro & Pinto, 2015; Lillvist & Granlund, 2010; Simeonsson, 2006).

Special support should focus on how to improve children's core engagement in everyday activities rather than solely on decreasing their hyperactive behaviour. Improved core engagement might lessen the impact of hyperactivity on children's learning. Accordingly, our results imply that even if all children in Swedish preschools have access to the same preschool activities, their level of core engagement varies depending on behavioural characteristics of the child and on contextual factors. Contextual factors, such as positive peer‐to‐child interaction and teacher responsiveness, seem to improve core engagement over time, which in turn have been found to improve children’s learning and academic achievement (Cadima et al., 2015; Ladd, & Dinella, 2009; Searle et al., 2013; McClelland et al., 2013), especially for children with hyperactive behaviour.

### Gender as a predictor of core engagement and hyperactive behaviour

Our findings indicate differences in the trajectories in core engagement and hyperactive behaviour for boys and girls. A steeper decrease in hyperactive behaviour over time is found for girls than for boys. Our results are consistent with the longitudinal studies by Sawyer et al. (2015) and Searle et al. (2014), which showed gender differences in engagement behaviour, behavioural difficulties, and later well‐being and learning outcomes. In preschool, boys with high levels of hyperactivity/inattention showed significantly lower engagement than girls with high levels of hyperactivity/inattention. These results may indicate a faster development of cognitive self‐regulation among girls (see Matthews, Ponitz, & Morrison, 2009). In our sample, a steeper increase in core engagement was associated with a decrease in hyperactive behaviour, especially for boys. These results suggest the importance of teachers planning and organizing preschool activities so as to improve core engagement for children displaying hyperactive behaviour, especially for boys.

The main message from our study is, however, that regardless of trajectories in core engagement and hyperactive behaviour, for boys and girls, an increase in hyperactive behaviour is associated with a decrease in core engagement. Low initial engagement and less increase in engagement with time might serve as indicators when identifying children in need of special support. The results support the argument that it is essential to design appropriate interventions to improve overall engagement in natural settings such as preschool (see Hughes, 2010; Phillips and Meloy, 2012) to facilitate future learning in school.

In addition, the present findings support previous studies showing that behaviour problems in early childhood are linked to lower levels of inhibitory control and lack of flexibility (Allan et al., 2015; Schoemaker et al., 2013), both inhibitory control and flexibility have also been related to engagement (Williford, Maier, et al., 2013). However, the results of this study did not find core engagement to be a significant predictor of change in hyperactive behaviour, and hyperactive behaviour at T1 was not a significant predictor of change in core engagement. Thus, trajectories in core engagement and hyperactive behaviour are at least partly independent. Therefore, when a child is exhibiting both low engagement and high levels of hyperactive behaviour, this cannot automatically be seen as two sides of the same problem. In other words, interventions for these children need to target core engagement as well as hyperactive behaviour and to consider characteristics of the child as well as contextual factors such as peer‐to‐child interaction and teacher responsiveness.

### Social interactions as predictors of core engagement and hyperactive behaviour

In contrast to the study by Fuhs et al. (2013), our findings indicate that teacher responsiveness predicts trajectories in core engagement but not hyperactive behaviour. However, teacher responsiveness was a significant predictor of hyperactive behaviour at T1 and for core engagement at T1. Teacher responsiveness may be indirectly associated with a change in hyperactive behaviour via child engagement. By organizing enjoyable activities and using responsive communication with the children, teachers might improve core engagement for children with hyperactive behaviour, which in turn decreases their behavioural difficulties. A longitudinal study by Searle et al. (2013) found that a positive child–teacher relationship was indirectly related to a negative association between children's engagement and inattention/hyperactive behaviour. A similar indirect pattern was found in our previous cross‐sectional study in Swedish preschools (Sjöman et al., 2016), where teacher responsiveness was a significant mediator between hyperactive behaviour and core engagement, explaining 33% of the variance. However, children's hyperactive behaviour can also be perceived by teachers as a source of stress, especially if the teacher and child are exposed to each other regularly over an extended period (Greene et al., 2002). This could, in turn, increase teacher–child conflicts (Mejia & Hoglund, 2016). Consequently, the association between teacher responsiveness and trajectories in core engagement may reflect both the contextual contribution of teachers' responsiveness, such as by the use of positive feedback and positive emotional tone, and that teachers, over time, might interact less frequently with children displaying hyperactive behaviour. Such possible child effects on teachers need to be studied in future research focusing on engagement for children with hyperactive behaviour.

Our findings indicate that positive peer‐to‐child interaction predicts high core engagement at T1 and an increase in core engagement over time. The results imply that a child's core engagement in activities with other children increases the likelihood that other children perceive the child as someone who can initiate, maintain, and end play in a socially accepted manner. To profit from the effects of peer‐to‐child interaction preschool teachers may have to focus more strongly on free play and how to enhance positive peer interaction in free play.

Compared with their peers, children in need of special support due to behavioural difficulties tend to spend less time highly engaged in preschool activities than other children, especially in free play activities which place high demands on their self‐regulation skills (Coplan et al., 2001; Guralnick et al., 2010). This study shows that positive peer‐to‐child interaction could lead to a decrease in hyperactive behaviour. Data for this study were collected in Swedish preschools. In Swedish preschools, children spend a high proportion of time (more than 50%) in free play and child‐initiated activities (Åström, Björck‐Åkesson, Sjöman, & Granlund, 2020). This implies that interventions aimed to improve positive peer relations, such as peer acceptance, number of mutual friendships, and proximity to others, to reduce children's behavioural problems in Swedish preschools (Witvliet et al., 2009).

### Study limitations and future directions

Some limitations should be considered when interpreting the findings from this study. First, even if our sample size (*n* = 203) was sufficient for applying multivariate models like LGC modelling, it is still relatively small (Boomsma & Hoogland, 2001). Additionally, all measures are reported by the same source of information (preschool staff), so there is a shared variance that partly can explain the relationships among variables. Also, the environment for some of the children changed over time and therefore also who rated child functioning. This may have biased the results in that children could have been assessed differently by different sources of information. Another possible source of bias concerns that possible teacher expectations on children in need of special support may have affected their ratings of the children. Children in need of special support affect their environment, as all children do (see Sameroff & Fiese, 2000). How children with, for example, hyperactive behaviour are perceived by staff may thus have influenced how the teachers rated these children's engagement. We were not able to fully control for these sources of bias in this study. However, previous findings using observations rather than proxy ratings have pointed in the same direction concerning the negative relationship between engagement and behavioural difficulties (Fuhs et al., 2013; Spivak & Farran, 2016), which supports our results.

Further, for some children the group size and the number of children per adult grew with age because of change of units. Age, in particular, may affect the trajectories of hyperactive behaviour and core engagement. Children probably will become less hyperactive when self‐regulation skills develop with age and children might stay highly engaged in more complex activities with age. However, the fact that we measure core engagement using items not strongly related to chronological age partly controls for the effects of age. That some children continue to exhibit high levels of hyperactive behaviour as they age also supports our interpretation of the results.

Overall, our results suggest that teacher responsiveness and positive peer‐to‐child interaction predict trajectories of children's core engagement, whether or not the children display hyperactive behaviour. Interventions focussing on peer‐to‐child interaction and teacher responsiveness and how to improve children's core engagement may thus provide positive effects for a whole preschool class. To get a deeper understanding of the mechanisms driving the relationship between social interactions and children's behaviour, further investigation needs to explore directional associations and reciprocal processes over time, between the child's positive and negative behaviours and peer‐to‐child interaction and teacher responsiveness.

## Conflict of interest

All authors declare no conflict of interest.

## Author contributions

Madeleine Sjöman, Ph.D. (Data curation; Formal analysis; Writing – original draft). Mats Granlund (Conceptualization; Project administration; Supervision; Writing –review & editing). Anna Karin Axelsson (Formal analysis; Methodology; Writing – review & editing). Lena Almqvist (Supervision; Writing – review & editing). Henrik Danielsson (Formal analysis; Methodology; Writing – review & editing).

## Data Availability

The data that support the findings of this study are available from the corresponding author upon reasonable request.
